# Identification of potential immunomodulators from *Pulsatilla* decoction that act on therapeutic targets for ulcerative colitis based on pharmacological activity, absorbed ingredients, and in-silico molecular docking

**DOI:** 10.1186/s13020-022-00684-7

**Published:** 2022-11-24

**Authors:** Li-rong Deng, Qian Han, Min Zou, Fang-jun Chen, Chang-yin Huang, Yi-ming Zhong, Qian-yan Wu, Brian Tomlinson, Yan-hong Li

**Affiliations:** 1 School of Medicine, South China University of Technology, Guangzhou, China; 2 Faculty of Medicine, Macau University of Science and Technology, Taipa, Macau China

**Keywords:** BTWT, Ulcerative colitis, Molecular docking, S1PR1, JAK3/STAT3, PD-1/PD-L1

## Abstract

**Background:**

*Pulsatilla* decoction (Bai-Tou-Weng-Tang, BTWT) is a classic formula prescription of a traditional Chinese medicine that is used to treat ulcerative colitis (UC). However, its active components and underlying mechanism of action remain unclear. In the present study, we aimed to identify potential immunomodulators from BTWT that act at therapeutic targets for UC.

**Methods:**

The protective effects of BTWT granules were examined in mice with colitis induced by dextran sulfate sodium. The absorbed components of BTWT were identified using LC-MS, and selected protein targets of these components in UC were investigated using molecular docking.

**Results:**

Oral administration of BTWT granules significantly alleviated disease severity and colon shortening, and inhibited the inflammatory response in mice with chronic colitis. In these mice, 11 compounds from the BTWT granules were detected in the serum and/or colon. The molecular docking study demonstrated that compounds from Radix *pulsatillae*, such as anemoside A3, interacted with STAT3 and S1PR1; compounds from Rhizoma *coptidis* and/or Cortex *phellodendri*, such as palmatine, interacted with JAK3, PD-1, and PD-L1; and components of *Cortex fraxini* such as aesculin interacted with S1PR1, JAK3, STAT3 and PD-L1. Further *in-vitro* experiments showing that the compounds inhibited TNF-α and IL-6 production and STAT3 activation in RAW 264.7 cells suggested that these compounds have immunomodulatory activities.

**Conclusion:**

We revealed for the first time that 11 absorbed ingredients from BTWT were immunomodulators against therapeutic targets for UC. These findings suggest that the identified compounds are the active components of BTWT, and the identified protein targets underlie the mechanism of action of BTWT against UC.

**Supplementary Information:**

The online version contains supplementary material available at 10.1186/s13020-022-00684-7.

## Introduction

*Pulsatilla* decoction, also named Bai-Tou-Weng-Tang (BTWT) in traditional Chinese medicine (TCM), has been used as a classic formula prescription in China for treating human diseases caused by bacteria for thousands of years [1]. The use of BTWT was first recorded in Zhang Zhongjing’s Treatise on Febrile Diseases, a famous classical book of TCM in China. In TCM theory, ulcerative colitis (UC) occurs mainly due to an excess of damp-heat, and BTWT functions by clearing away internal heat and detoxifying, cooling blood, and stopping bleeding. Therefore, BTWT is widely used for treating UC in TCM, and its efficacy has been verified by clinical observations and in experimental models of UC [2–4]. However, prior to this study, it was largely unknown which active components of BTWT are responsible for its immunomodulatory activity. As such, its use as a standard therapy is restricted and it can only be used in specific hospitals that give instructions in the theory of TCM.

BTWT is composed of one monarch herb, Radix *pulsatilla* (*R. pulsatilla*, the dried radix of *Pulsatilla chinensis* (Bunge) Regel) (15 g); two minister herbs, Rhizoma *coptidis* (*R. coptidis*, the dried rhizome of *Coptis chinensis* Franch.) (6 g); Cortex *Phellodendri* (*C. phellodendri*, the dried cortex of *Phellodendron chinense* C.K.Schneid.) (12 g), and one guiding herb, Cortex *fraxini* (*C.* *fraxini*, the dried cortex of *Fraxinus chinensis* Roxb.) (12 g) (Wang et al., 2019). The compounds isolated from *R. pulsatilla* include triterpenoid saponins, phytosterone, and anthocyanins [5]. Triterpenoid saponins are the major bioactive compounds of *R. pulsatilla*; anemoside B4 (AB4) is present in the highest amount followed by anemoside A3 (AA3) and 23-hydroxybetulinic acid (23-HA) [6]. *R. coptidis* and *C. phellodendri* are rich in alkaloids such as berberine, palmatine, coptisine, and epiberberine [7, 8]. *C.* *fraxini* contains several chemicals, of which aesculin and aesculetin are the main active ingredients. Several recent studies have provided clues about the components of BTWT that mediate efficacy in the treatment of UC. For example, AB4, berberine, palmatine, coptisine, and aesculin can ameliorate dextran sulfate sodium (DSS)-induced colitis [9–14]. Thus, these compounds might mediate the effects of BTWT in UC; however, no studies have systemically investigated the active components of BTWT. In this study, after verifying the preventative and therapeutic effects of BTWT in two mouse models of UC, we investigated the pharmacological targets of the absorbed ingredients that originated from the four component herbs of BTWT.

Several pathways are involved in the pathogenesis of UC, and components of these pathways are either targets of approved drugs or under investigation as potential therapeutic targets in UC. For example, blocking the Janus tyrosine kinase (JAK)/signal transducer and activator of transcription (STAT) pathway by inhibitors of JAK3 or STAT3 inhibits the production of pro-inflammatory cytokines, and thus such inhibitors are used as new treatments for UC [15]. Targeting the sphingosine-1-phosphate receptor modulator (S1PR) with ozanimod, which blocks lymphocyte egress from the lymph nodes and prevents lymphocyte recirculation, is another novel strategy for treating UC [16]. In addition, programmed cell death protein 1 (PD-1) and its ligand PD-L1 play a key role in reducing harmful immune responses and maintaining immune tolerance [17]. Blockade of this pathway in humans can induce serious side effects that resemble UC [18, 19]. Lastly, nuclear factor erythroid 2-related factor 2 (Nrf2) is a stress response transcription factor that regulates gastrointestinal function and is a promising candidate for preventing UC [20].

*In-silico* molecular docking uses computer pattern recognition and optimization technology to search for molecules from a three-dimensional structure database that geometrically and chemically match specific drug targets [21, 22]. Molecular docking can help elucidate the interactions and binding mechanisms between a protein target and its ligands [23, 24]. It has been used to identify ligands for some active components from plants materials [25, 26]. The objective of this study was to use *in-silico* docking to identify potential immunomodulators from BTWT, using JAK3, STAT3, S1PR1, PD1, PD-L1, and Nrf2 as protein targets.

## Materials and methods

### Chemicals and reagents

**Fig. 1:**
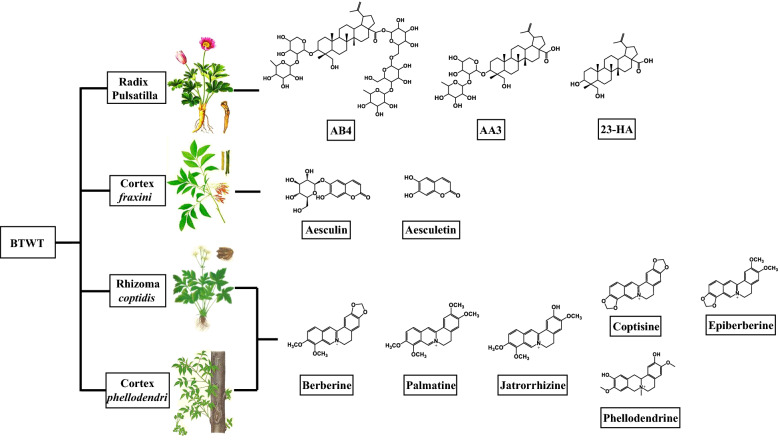
The label components of the four component herbs of BTWT

The concentrated granules (with a concentrate to dried herb ratio of 10:0.9) of four component herbs of BTWT, *R. pulsatilla, R. coptidis*, *C. phellodendri*, and *C. fraxini* (lot numbers 8055671, 9010401, 1612619, and 7122571, respectively), were purchased from Guangdong Yifang Pharmaceutical Co., Ltd (Foshan, China). The structures of representative components from the four herbs are shown in Fig. 1. AB4, AA3, 23-HA, aesculin, aesculetin, palmatine, jatrorrhizine, coptisine, epiberberine, and phellodendrine were purchased from Yuanye Bio-Technology Co., Ltd at the highest available purity (98%) (Shanghai, China). Berberine hydrochloride was purchased from Shenzhen ChemStrong Scientific Co., Ltd (Shenzhen, China) at the highest available purity (95%). DSS was purchased from MP Biomedicals (MW; 36,000 − 50,000, Solon, OH, USA). The stool bleeding test kit was purchased from BaSO Bio-Technology Co., Ltd (Zhuhai, China). Lipopolysaccharide (LPS) was purchased from Sigma-Aldrich. Deionized water was purified using a Millipore water purification system (Millipore, Milford, MA, USA). All other reagents used were of analytical grade.

### Analysis of the components of BTWT granules by liquid chromatography-mass spectrometry (LC–MS)

Granules of the four herbs were accurately weighed (35.1 mg *R. pulsatilla,* 26 mg *R.* *coptidis,* 26 mg *C. phellodendri* and 15.6 mg *C.* *fraxini*) and dissolved in 2 mL of sterile water using an ultrasonicator. The final concentration of BTWT solution was 51.35 mg/ml. The solution was freshly prepared before analysis. For sample extraction, 0.59 ml methanol was added to 10 μl BTWT solution and vortexed for 10 min before centrifugation and evaporation. The residue was reconstituted in 1 ml mobile phase. The concentration of the sample for LC–MS detection was 513.5 μg/ml. A mixture of standard stock solution containing the 11 compounds was prepared in methanol at a final concentration of 3000 ng/ml AB4, 300 ng/ml AA3, 300 ng/ml 23-HA, 1200 ng/ml berberine, 1200 ng/ml palmatine, 1200 ng/ml coptisine, 800 ng/ml jateorhizine, 1500 ng/ml epiberberine, 300 ng/ml phellodendrine, 900 ng/ml aesculin, and 450 ng/ml aesculetin. Quantification was carried out on an ultra-high-performance liquid chromatograph (UPLC) with triple quadrupole tandem mass spectrometer (MS). The UPLC analysis was performed using a Shimadzu Nexera X2 LC-30AD (Shimadzu, Japan) system coupled to an AB Sciex 4500 QTRAP mass spectrometer (AB Sciex, USA) equipped with an electrospray ionization interface (ESI). Chromatographic separation was achieved on a Kinetex C18 column (Phenomenex, USA) (2.1 mm i.d. × 100 mm, 2.6 μm) equipped with a column heater. The mobile phase consisted of solvent A (0.1%, v/v, of formic acid in water) and solvent B (acetonitrile). The flow rate was 0.3 ml/min, the injection volume was 4 μL, and the column temperature was maintained at 35 ℃. Mass spectrometry data were acquired in multiple reaction monitoring mode and negative ESI mode.

### Experimental animals

Male C57BL/6 mice, 8 –11 weeks old, weighing 23–25 g, obtained from Hunan Silaike Jingda Laboratory Animal Co Ltd (Hunan, China) were used in this study. The animals were housed at a controlled temperature (24 ± 2 °C) and with 12 h/12 h light/dark cycles. They were fed standard mice chow pellets and had access to filtered water. All animal care and experimental procedures were approved by the Animal Care and Use Committee at South China University of Technology (SCUT) (approval number: 2017004).

### Induction of colitis and drug treatment

Acute colitis was induced by giving mice 2% DSS for 5 days and followed by 3 days of water. Chronic colitis was induced by giving mice 3 cycles of 2% DSS following a protocol previously described [27]. After 5 days or the first round of 4 days DSS administration, all mice developed symptoms of colitis and were randomly divided into two groups with five mice in each group; one group that received DSS-only, and one group that received BTWT (531.3 mg/kg). The dose equivalent to BTWTs granule was determined according to the human clinical equivalent dose of crude herb (45 g/70 kg for humans) and converted to 5.85 g/kg of crude herb for mice, and finally calculated as 513.5 mg/kg concentrated granules. All drug solutions were freshly prepared daily and orally administrated based on the actual weight of the mice (0.1 ml/10 g). The administration of drugs started on day 0 and lasted for 8 days for the acute model. For the chronic model, drug administration started on day 5 and lasted for 14 days. One group of mice designated as the water group received filtered water alone throughout the acute or chronic experimental procedure.

### Determination of clinical score, colon length, and colon weight

Body weight, stool consistency, and occurrence of occult blood or the presence of gross blood per rectum were determined according to previously described criteria [27–29]. Weight loss, stool consistency, and stool bleeding sub-scores were added, resulting in a total clinical score ranging from 0 (healthy) to 8 (maximal activity of colitis). At the end of treatment, the animals were sacrificed by cervical dissociation, and the entire colon from the cecum to the anus was removed. The colon length and colon weight were measured before the colon was divided into several segments for later biochemical examination.

### Analysis of absorbed BTWT ingredients by LC–MS analysis

Plasma (200 μL) and colon (70 mg) samples were taken from mice with acute colitis 1 h after the last intragastric administration of BTWT (531.5 mg/kg per day for 8 days), and the distribution of the 11 BTWT ingredients in these samples were determined by LC–MS analysis as described above.

### Simulation setup and docking parameters

All *in-silico* simulations were executed using the software packages ChemDraw (version 16.0.082) and Molecular Operating Environment (MOE, version 2010.10). Seven compounds that are reported as respective ligands of the six selected protein targets were used as standards (positive controls). These compounds were as follows: the JAK3 inhibitor tofacitinib [30]; SD-36, which is a small-molecule degrader of STAT3 [31]; the S1PR1 endogenous ligand S1P [32] and the S1PR1 antagonist ML056 (W146); ML385, which blocks the activity of Nrf2 [33]; nivolumab, which is a monoclonal antibody against PD-1 [34]; and BSM-202, which is a small-molecule compound that potently blocks binding of PD-L1 to PD-1 [35]. The chemical structures of these positive-control ligands and selected components of BTWT were constructed using ChemDraw then imported into the MOE database file after 3-D protonation and energy minimization with the MMFF94x force field using default parameters.

The positive-control ligands for the docking simulations were created using MOE Builder and manually inserted into the active site of the six proteins extracted from the respective PDB files. The docking experiments were performed 100 times for each compound, and the 30 lowest energy conformations (poses) were recorded in order of increasing potential energy of the conformation in kcal/mol. For specific binding analysis, the best conformation for each compound was visualized and evaluated for analyses of binding modes between atoms or moieties of the ligands and specific residues of the target proteins [36].

### Splenocyte culture and cell viability assay

SplenocyteS isolated from the spleen of mice were cultured in RPMI 1640 medium supplemented with 10% fetal bovine serum (FBS) and 100 U/ml each of penicillin, and streptomycin (Gibico, Invitrogen, Carlsbad, CA, USA) overnight. To evaluate the cell viability, 2 × 10^5^ splenocytes cultured in 96-well plates were treated with 1 µM of 11 components of BTWT for 24 h and then submitted to cell viability assay by mitochondria-dependent reduction of thiazolyl blue tetrazolium bromide (MTT) (Sigma-Aldrich). All experiments were repeated in quadruplicate in different intervals.

### RAW 264.7 cell culture and drug treatment

RAW 264.7 cells were used as an in vitro model to test the effects of the 11 adsorbed BTWT compounds on cytokine production. RAW 264.7 cells were cultured at 1.0 × 10^6^ cells/ml in 96-well plates in RPMI-1640 media (Gibco, Carlsbad, CA USA) containing 10% FBS (Gibco, Carlsbad, CA USA. Cells were pretreated with 1 μM tofacitinib, AB4, AA3, 23-HA, berberine, epiberberine, palmatine, coptisine, jateorhizine, phellodendrine, aesculin, or aesculetin for 22 h, and then incubated with 1 μg/ml LPS for 2 h at 37 °C in a humidified atmosphere with 5% CO_2_. At the end of the treatment, the supernatant from RAW 264.7 cell culture was collected and stored frozen (−80 °C) for analysis by enzyme-linked immunosorbent assay (ELISA) assay.

### mRNA extraction and quantitative real-time reverse-transcription polymerase chain reaction (RT-qPCR)

Total RNA from the colon or RAW 264.7 cells was isolated using TRIzol reagent (Invitrogen; ThermoFisher Scientific, Waltham, MA, USA) and subjected to mRNA purification using lithium chloride precipitation as previously described [28]. Reverse transcription was performed with a cDNA synthesis kit (TaKaRa Biotech, Japan). The mRNA expression of interleukin-17 (IL-17), interleukin-6 (IL-6), tumor necrosis factor alpha (TNF-α), and the internal control glyceraldehyde 3-phosphate dehydrogenase (GAPDH) was measured by RT-qPCR using the 7500 real-time PCR system (Applied Biosystems; ThermoFisher Scientific, Waltham, MA, USA). Mouse mRNA primer sequences were the same as those used in our previous study [29]. The mRNA expression level in the water-treated control group was set as 1, and the mRNA expression levels in the drug-treated mice and cells were compared with those of the water-treated group.

### ELISA

The levels of TNF-α and IL-6 in the cell culture supernatant of RAW 264.7 cells and splenocytes were measured using mouse Quantikine ELISA kits (TNF-α: Cat. No. 430904, IL-6: Cat. No. 431304, Biolegend, San Diego, CA, USA) according to the manufacturer’s protocol. All samples were analyzed in duplicates. The concentrations of TNF-α and IL-6 were calculated by standard curves and expressed as pg/ml.

### Western blotting

RAW 264.7 cells were homogenized in RIPA buffer and the protein concentration was determined using a BCA protein assay kit (Biosharp, Anhui, China). The immunoblot was then incubated with primary antibodies against phospho-STAT3 (pSTAT3; Cell Signaling Technology, Danvers, MA, USA; catalog number 9145 s), STAT3 (Proteintech, Rosemont, IL, USA; catalog number 60199–1-Ig), and β-actin (Affinity Biosciences, Jiangsu, China; catalog number T002-50). The obtained chemiluminescence signals were analyzed with ImageJ software.

### Data analysis

All data are presented as the means ± standard deviation (SD) or means ± standard error of the mean (SEM) of at least three biological replicates. One-way or two-way ANOVA coupled with Dunnett’s post-test was used to test for statistically significant differences in the mean values between each treatment. Statistical analyses were performed using Prism 6 software for Windows (GraphPad Software, Inc., San Diego, CA, USA). *P*-values of 0.05 or less were considered statistically significant.

## Results

### Identification of the components of BTWT granules by LC–MS

**Fig. 2:**
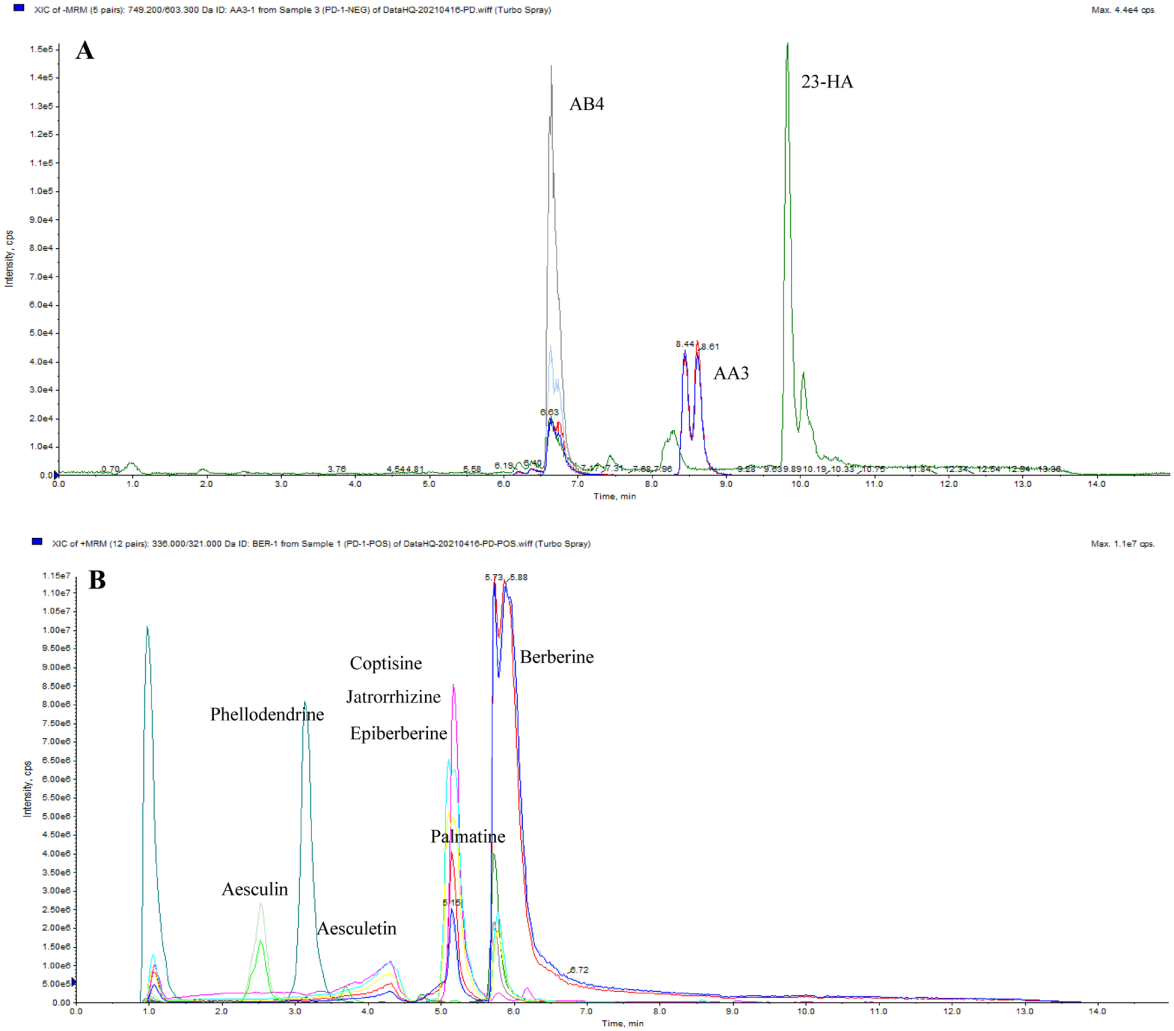
A total ion chromatogram of a BTWT sample in negative **A** and positive **B** mode

As shown in Fig. 2A, B LC-MS was used to analyze the components of BTWT granules. The following standard substances were used for the analysis: AB4, AA3, 23-HA, berberine, epiberberine, palmatine, coptisine, jateorhizine, phellodendrine, aesculin, and aesculetin. The existence of the standard substances in BTWT granules was confirmed by comparing the peak retention times of the 11 standard substances with those of the isolated BTWT components.

### The in-vivo anti-inflammatory activity of BTWT against experimental colitis in mice

**Fig. 3:**
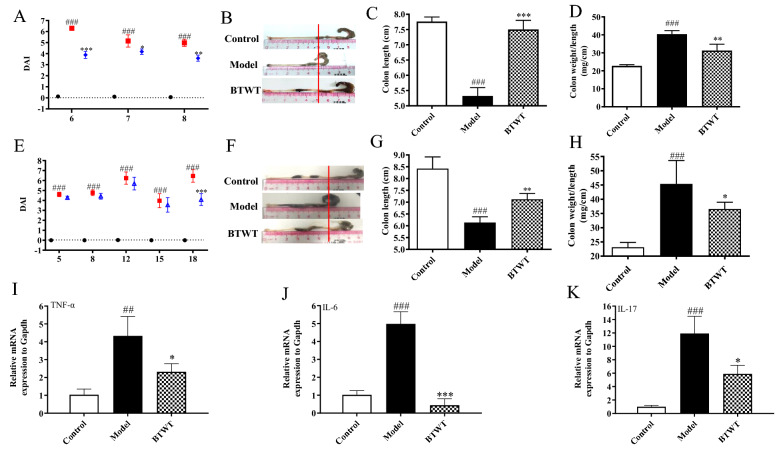
The anti-inflammatory effects of BTWT in mice with DSS-induced colitis. The preventative effects of BTWT on the severity of acute colitis was demonstrated by assessing the DAI **A**, the colon length (**B** and **C**), and the relative colon weight to length **D** after the mice were sacrificed at the end of the 8 days treatment. The therapeutic effects of BTWT on the severity of chronic relapsing colitis was demonstrated by assessing the DAI (**E**), the colon length (**F** and **G**), and the relative colon weight to length **H** after mice were sacrificed at the end of the 14 days treatment. The mRNA expression of TNF-α **I**, IL-6 **J**, and IL-17 **K**, and the housekeeping gene GAPDH in colon tissue was determined by RT-qPCR. ^##^ P < 0.01 and.^###^ P < 0.001 compared with the water control group; *P < 0.05, **P < 0.01, and ***P < 0.001 compared with the DSS group (n = 5 mice per treatment group)

In the 8 days acute colitis model, mice exposed to 2.0% DSS for 5 days developed signs of colitis, as evidenced by a significant increase in the clinical disease activity index (DAI) in all the groups on days 6, 7, and 8, accompanied by a decrease in colon length (colon shortening) and an increase in the relative weight of the colon (indicating colonic swelling) compared with water-treated control animals (Fig. 3**A–D**). Compared with the DSS-treated control, the preventative administration of 531.5 mg/kg BTWT significantly reduced the DAI at the three tested times (Fig. 3**A**) and inhibited the DSS-induced colon shortening and swelling (Fig. 3**B–D**).

In the 18 days colitis model, mice exposed to three cycles of 2.0% DSS for 4 days had a significant increase in the DAI at all time points compared with water-treated control mice (Fig. 3E). Compared with the DSS-treated control, the therapeutic administration of 531.5 mg/kg BTWT from day 5 significantly reduced the DAI at the end of treatment (day 18). Furthermore, the colon shortening and swelling caused by three cycles of DSS challenge were significantly antagonized by BTWT treatment (Fig. 3F–H). Further, BTWT treatment significantly reduced the DSS challenge-associated increase in the mRNA levels of TNF-α, IL-6, and IL-17 in the colon of mice with chronic relapsing UC (Fig. 3I–K).

### The absorbed constituents from orally administered BTWT granules

**Fig. 4:**
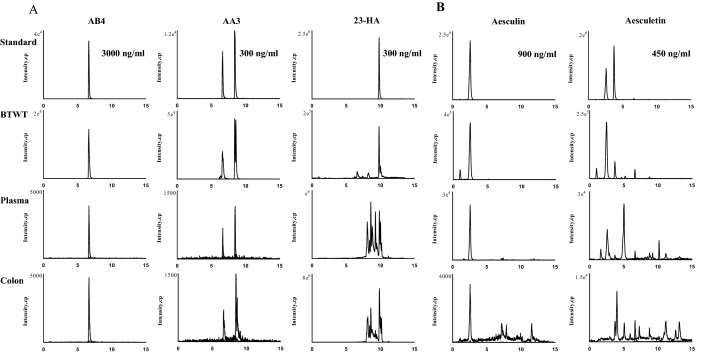

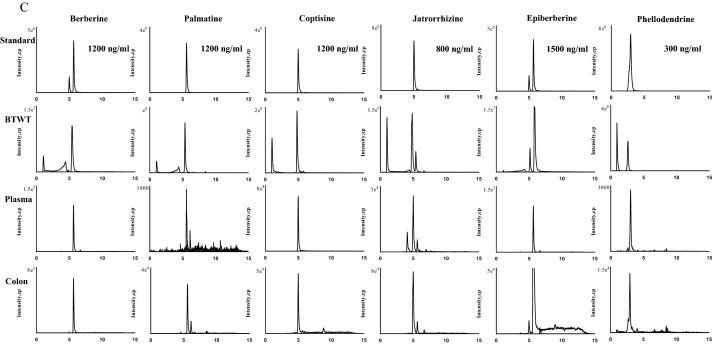
Representative UPLC–QQQ-MS chromatograms of the identified components from *R. pulsatilla ***A**, *C. fraxini* **B**, *C. phellodendri*, and *R. coptidis* **C** in mouse plasma and colon samples after oral administration of 531.5 mg/kg BTWT (n = 5 mice per treatment group)

Individual ingredients known to constitute the raw materials of the BTWT were analyzed as standards using the same chromatogram conditions to confirm that the peaks obtained from the UPLC analysis represented recognized constituents of BTWT (Fig. 4). A total of 11 compounds identified by UPLC-Q/TOF–MS analysis were assigned to an original herbal medicine known to be present in BTWT (Fig. 1). To define the absorbed chemical constituents, mass spectrometry data of standard chemicals was compared with mass spectrometry data of the absorbed compounds distributed in the circulation and colon tissue of mice. As demonstrated in Fig. 4, three compounds, 23-HA, epiberberine, and aesculetin, were only detected in mice colon samples. The remaining components, namely, AB4, AA3, berberine, palmatine, coptisine, jateorhizine, phellodendrine, and aesculin, were detected in both colon and plasma samples (Fig. 4).

### Binding of BTWT constituents to JAK3

**Fig. 5:**
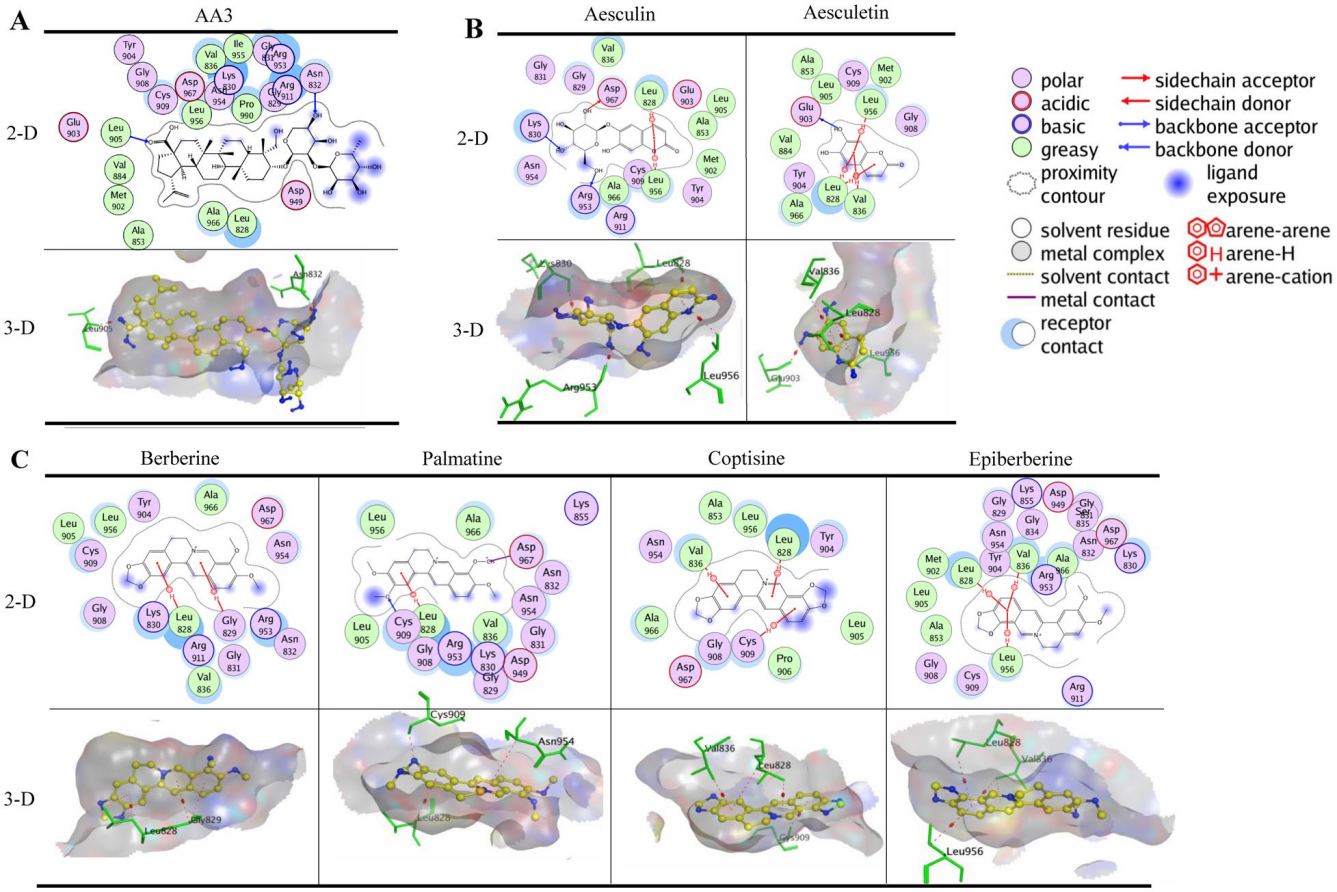
Molecular docking results of AA3 **A**, aesculin, aesculetin **B**, berberine, palmatine, coptisine, and epiberberine **C** in the binding sites of JAK3 with the lowest calculated binding energy conformations. In the 2D images, polar and nonpolar residues in the active site of JAK3 are shown in purple and green, respectively. Hydrogen bonds in the sidechain and backbone are shown in red and blue arrows, respectively; the arrows point to the H-acceptor. The π-H interactions are marked with a red line and an arene-H label. The metal contact interactions are shown with a purple line. In the 3D images, the carbon atoms of the compounds are shown in yellow, and other atoms (e.g., oxygen, nitrogen, and sulfur atoms, etc.) are shown in blue. The amino acid residues of the JAK3 proteins that interacted with the compounds are shown in green. The formation of hydrogen bonds and π-H and π-π interactions are shown by a red dotted line

As shown in Additional file 1: Fig. S1A, tofacitinib formed two hydrogen bonds with JAK3; Leu905 acted as a H-acceptor for the amine atom of the pyrrole moiety, and Glu903 acted as a H-donor for the pyrimidine moiety with the lowest calculated binding energy conformation value of -5.93 kcal/mol. This result was similar to results from two previous studies [37, 38]. In addition, the pyrrole moiety formed a π-H interaction with Leu828, and pyrimidine moiety formed two π-H interactions with Leu828 and Leu956 of JAK3. When docked to JAK3, all the tested compounds of BTWT achieved the lowest calculated binding energy conformation values ranging from −12.90 to −4.30 kcal/mol. As shown in Fig. 5A, only one component of *R. pulsatilla*, AA3, interacted with JAK3 in a way similar to that of tofacitinib; AA3 formed a hydrogen bond with Leu905; and Leu905 acted as an H-acceptor interacting with the oxygen atom of the ester group of AA3 (Fig. 5A). Notably, two components of *C. fraxini* formed numerous interactions with JAK3; these interactions also occur when tofacitinib is docked to JAK3. Specifically, the phenyl ring and/or the pyranoid ring of benzopyran-2-one of aesculin, aesculetin formed π-H interactions with Leu828 and/or Leu956 of JAK3 (Fig. 5B). Also, the hydroxyl groups of aesculetin formed a hydrogen bond with Glu903 of JAK3; Glu903 acts as a H-donor, similar to what is seen with tofacitinib binding (Fig. 5B). By contrast, four components of *R. coptidis* and *C. phellodendri*, namely, berberine, palmatine, coptisine, and epiberberine, formed π–H interactions with Leu828 of JAK3 through their phenyl ring and the pyridine ring of the isoquinoline ring (Fig. 5C).

### Binding of BTWT constituents to STAT3

The SH2 domain of STAT3 is the main target of small-molecule inhibitors. Hence, the pocket-type subdomain structures of STAT3 including the pTys705 binding sites (Lys591, Arg609, Ser611, and Ser613) were selected as binding pockets [39]. As shown in Additiional file 1: Fig. S1A, with the lowest calculated binding energy conformation value of −7.45 kcal/mol, SD-36 formed four hydrogen bonds with STAT3; Arg609, Ser611, Glu612, and Ser613 acted as H-acceptors for the interaction with oxygen atoms of the phosphate moiety of SD-36. SD-36 also formed a hydrogen bond with STAT3, and Glu638 acted as an H-donor for the amine atom of the indole ring. Moreover, one of the oxygen atoms of the phosphate moiety also formed a metal contact with Arg609. All tested compounds present in BTWT were docked to STAT3 and obtained the lowest calculated binding energy conformation values ranging from −8.54 to −2.53 kcal/mol. AB4 had the lowest energetic conformation value (−8.54 kcal/mol), and this was the only value that was comparable to that of SD-36 (−7.54 kcal/mol). Two *R. pulsatilla* components, AA3 and 23-HA, and one *C. fraxini* component aesculin formed hydrogen bonds with STAT3, which were also observed with SD-36 binding (Fig. 5A). Specifically, the hydroxyl groups of AA3 formed three hydrogen bonds with STAT3, with Ser611 and Sre613 as H-acceptors and Glu638 as an H-donor. In addition, 23-HA formed hydrogen bonds with STAT3; Arg609 and Ser611 acted as the H-acceptor for the ionized oxygen atom of the ester group, and Glu612 and Ser613 acted as the H-acceptor for the oxygen atom of the ester group.

### Binding of BTWT constituents to S1PR1

**Fig. 6:**
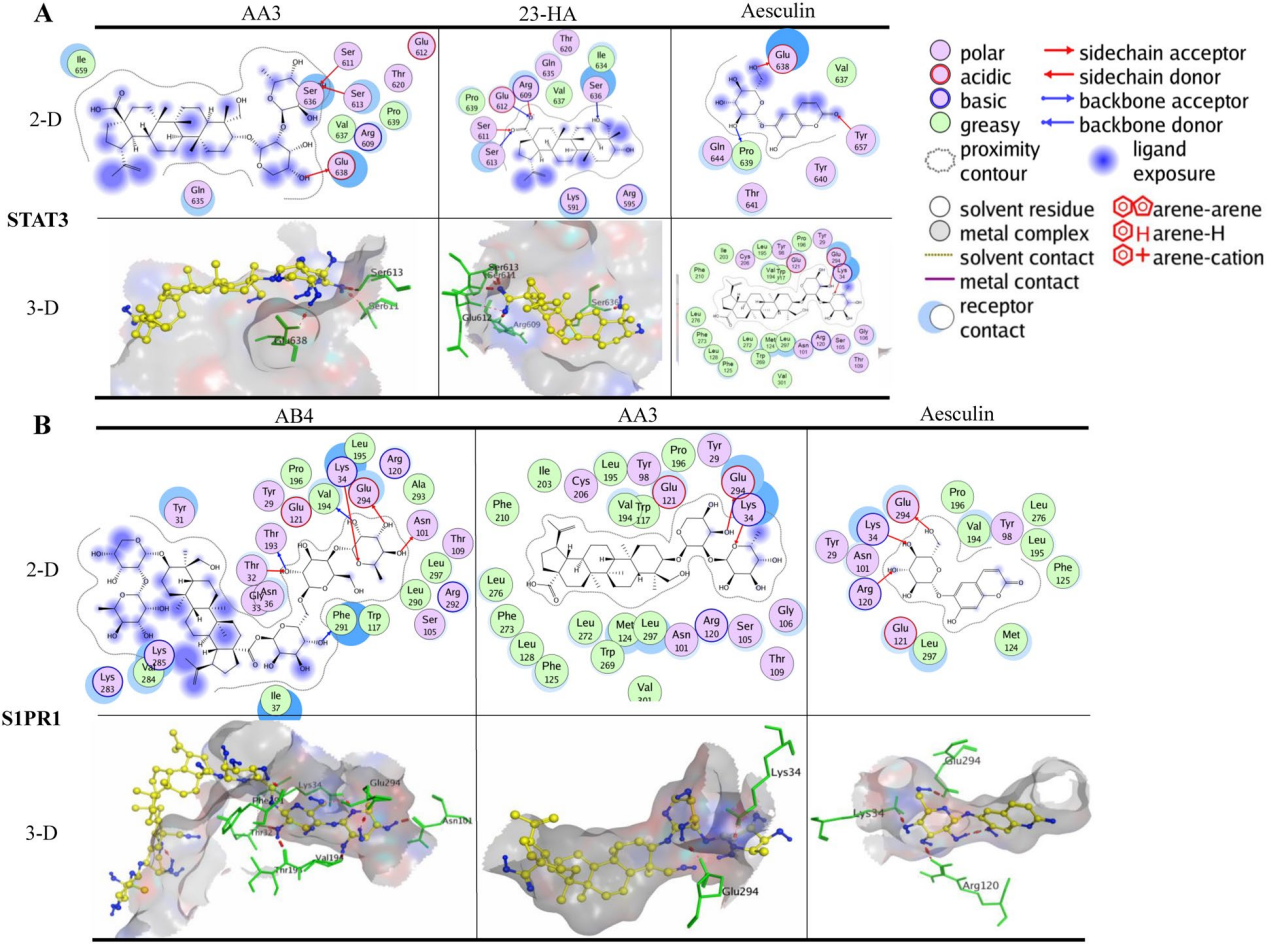
Molecular docking results of AB4, AA3, 23-HA, and aesculin in the binding sites of STAT3 **A** and S1PR1 **B** with the lowest calculated binding energy conformations. In the 2D images, the polar and nonpolar residues of the active site of STAT3 and S1PR1 are shown in purple and green, respectively. The hydrogen bonds in the sidechain and backbone are shown in red and blue arrows, respectively; the arrows point to the H-acceptor. The π-H interactions are marked with a red line. In the 3D images, the carbon atoms of the compounds are shown in yellow, and other atoms (e.g., oxygen, nitrogen, and sulfur atoms, etc.) are shown in blue. The amino acid residues of the STAT3 and S1PR1 proteins that interacted with compounds are shown in green. The formation of hydrogen bond interactions is shown with a red dotted line

The lowest calculated binding energy conformation value of S1P docked to the active site of S1PR1 was -12.90 kcal/mol, which is lower than that of ML056 (W146) (-10.6 kcal/mol). S1P formed hydrogen bonds with Asn101 of S1PR1 acting as the H-donor for the protonated amine of the alkyl chain, and with Glu294 of S1PR1 acting as the H-donor for the protonated amine hydroxyl group; this binding mode was also seen by Yuan et al*.* [40]. In contrast, the phosphate moiety of ML056 (W146) formed hydrogen bonds with Try29 and Arg120 and formed a metal contact with Arg120 (Additional file 1: Fig.S1A); this binding mode has been reported by several studies [41–43]. Docking of components contained in BTWT to S1PR1 gave the lowest calculated binding energy conformation values, ranging from -13.00 to -5.84 kcal/mol; AA3 had the lowest value. As shown in Fig. 6B, the oxygen atom of the glucopyranosyl or hydroxyl group of AB4, AA3, and aesculin formed hydrogen bonds with S1PR1 with Lys34 acting as an H-acceptor. This binding mode was also observed in the interaction of the two positive-control ligands and S1PR1. Furthermore, the hydroxyl groups of AB4, AA3, and aesculin formed hydrogen bonds with Glu294 of S1PR1 acting as the H-donor. The above two binding modes were also observed in the S1P–S1PR1 interaction (Additional file 1: Fig.S1A). These observations indicate that AA3, aesculin, and especially AB4 are highly likely to be potent S1PR1 modulators.

### Binding of BTWT constituents to Nrf2

Docking of ML385 to Nrf2 reached the lowest calculated binding energy conformation value of −7.11 kcal/mol, showing that a π-H bond interaction occurred between the benzodioxole group of ML385 and His20 of Nrf2 (Additional file 1: Fig. S1B). The docking simulation of selected components in BTWT to the active site of Nrf2 gave the lowest calculated binding energy conformation values, ranging from -9.14 to -4.66 kcal/mol. Unlike the positive-control ligand ML385, compounds from BTWT did not interact with residue His20 of Nrf2. However, interactions between compounds and other residues occurred; the protonated amine of the isoquinoline ring of palmatine and coptisine made a metal contact with Glu25. No interactions were formed between 23-HA, epiberberine, aesculin, and aesculetin and Nrf2.

### Binding of BTWT constituents to PD-1

**Fig. 7:**
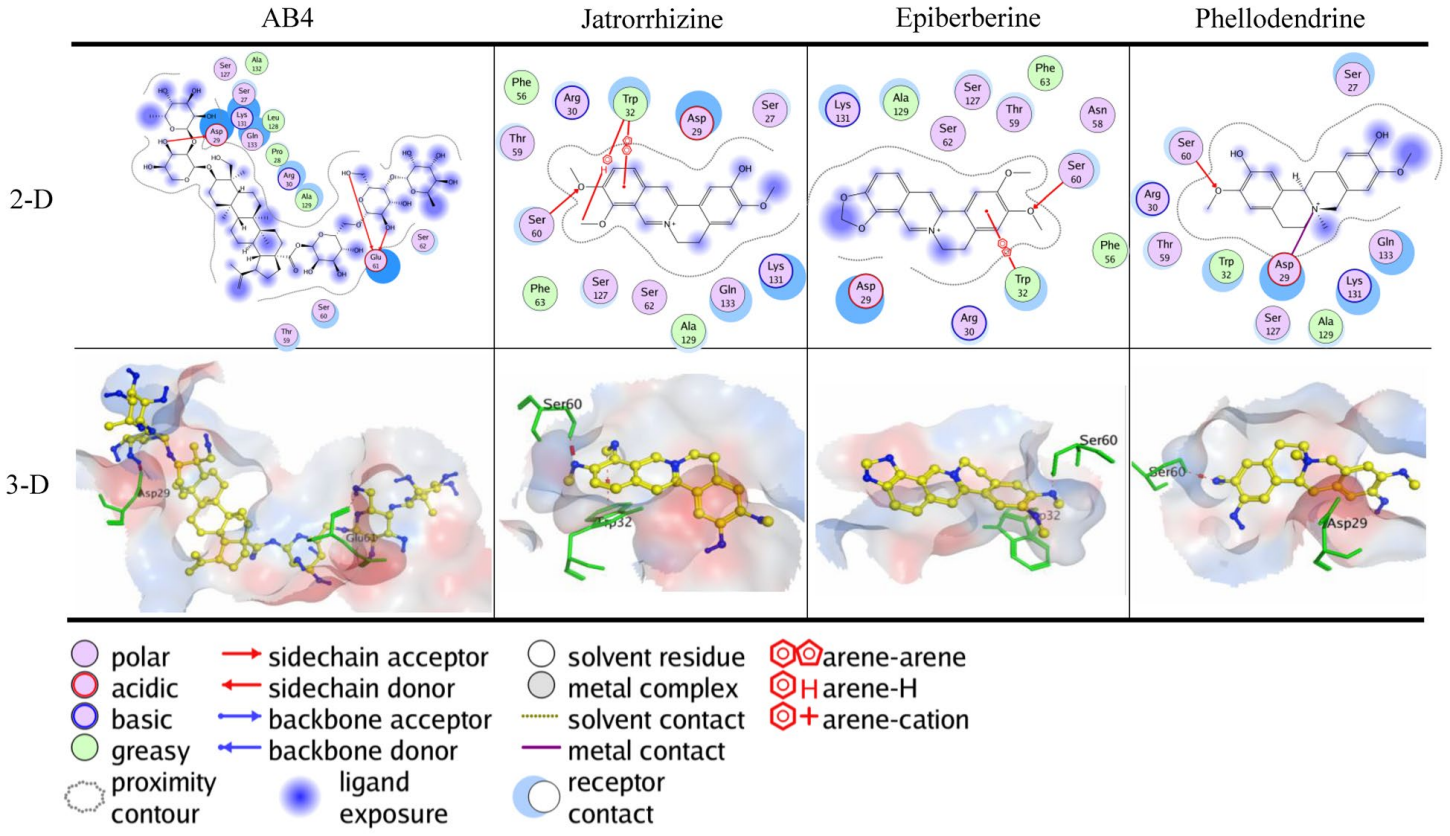
Molecular docking results of AB4, jatrorrhizine, epiberberine, and phellodendrine in the binding site of PD-1 with the lowest calculated binding energy conformations. In the 2D images, the polar and nonpolar residues of the active site in the PD-1 protein are shown in purple and green, respectively. Hydrogen bonds in the sidechain are shown by red arrows; the arrows point to the H-acceptor. The π-H interactions are marked with a red line and an arene-H label. The π-π interactions are marked with a red line and arene-arene label. The metal contact interactions are shown with a purple line. In the 3D images, the carbon atoms of the compounds are shown in yellow, and other atoms (e.g., oxygen, nitrogen, and sulfur atoms, etc.) are shown in blue. The residues of the PD-1 protein that interacted with the compounds are shown in green. The formation of hydrogen bonds and π-H and π-π interactions are shown by the red dotted line

Nivolumab formed three hydrogen bonds with PD-1, with Asp29 and Gln133 acting as H-acceptor for the hydroxyl group and Ser60 acting as H-acceptor for the ammonia atom of the amide group (Additional file 1: Fig.S1B). The lowest calculated binding energy conformation value was −6.49 kcal/mol. This observed binding mode of nivolumab with Asp29 has been reported in two previous studies [34, 35]. The docking simulation of selected components in BTWT to the active site of PD-1 gave the lowest calculated binding energy conformation values ranging from −6.83–−3.19 kcal/mol. As shown in Fig. 7, only one component of *R. pulsatilla*, AB4, interacted with PD-1 in a way that was similar to the PD-1–nivolumab interaction. This interaction involved the formation of a hydrogen bond between the hydroxyl group of AB4 and Asp29 acting as a H-acceptor. In addition, the oxygen atom of the methoxy group of jatrorrhizine, epiberberine, and phellodendrine formed hydrogen bonds with Ser60 of PD-1 acting as the H-acceptor.

### Binding of BTWT constituents to PD-L1

**Fig. 8:**
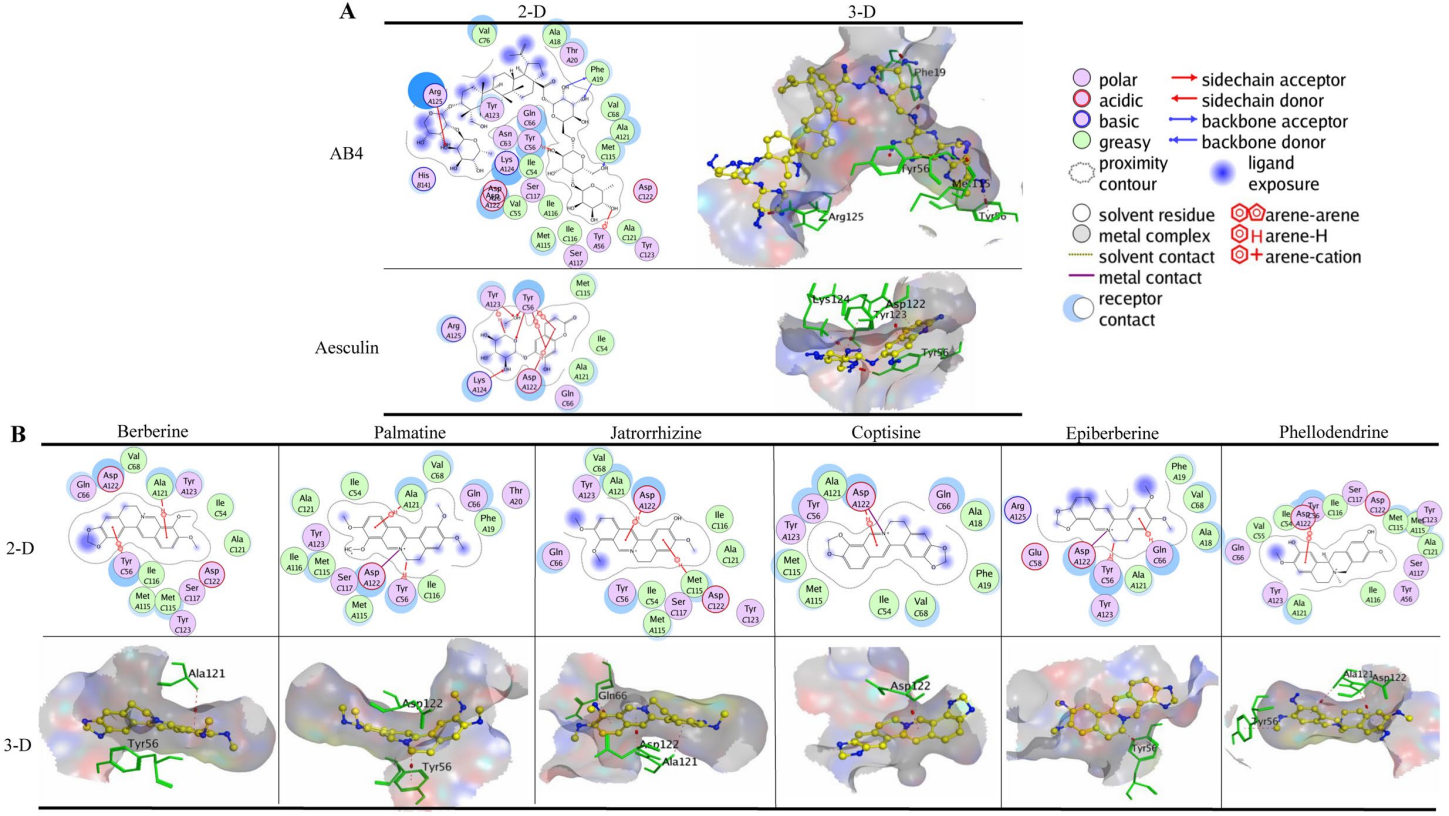
Molecular docking results of AB4, aesculin **A**, berberine, palmatine, jatrorrhizine, coptisine epiberberine, phellodendrine **B** with the binding site of PD-L1 with the lowest calculated binding energy conformation. In the 2D images, the polar and nonpolar residues of the active site of the PD-L1 protein are shown in purple and green, respectively. Hydrogen bonds in the sidechain are shown by the red arrows; the arrow points to the H-acceptor. The π-H interactions are marked with a red line and an arene-H label. The π-π interactions are marked with a red line and arene-arene label. The metal contact interactions are shown with a purple line. In the 3D images, the carbon atoms of the compounds are shown in yellow, and other atoms (e.g., oxygen, nitrogen, and sulfur atoms, etc.) are shown in blue. The residues of the PD-L1 protein that interacted with the compounds are shown in green. The formation of hydrogen bonds and π-H and π-π interactions are shown with a red dotted line

BMS-202 reached the lowest calculated binding energy conformation value of -8.92 kcal/mol after docking optimization. Four π-H bonds were formed between the phenyl ring of BMS-202 and residues GlnC66, AlaA121, AspC121, and AspA122 of PD-L1. Another π-H bond was formed between the carbon atom of the phenyl ring of BMS-202 and TyrA56 of PD-L1 (Additional file 1: Fig. S1B). Both of these interactions between BMS-202 and PD-L1 have been described in previous studies [44, 45]. In addition, the oxygen atom of the amide group of BMS-202 formed a hydrogen bond with LsyA124 acting as an H-acceptor, and the protonated amine formed a metal contact with AspA122 (Additional file 1: Fig. S2B). About half of the components of BTWT produced the lowest calculated binding energy conformation value that was similar or lower to that of BMS-202; for example, the values of AB4 and berberine were −11.28 and −6.32 kcal/mol, respectively. As shown in Fig. 8, the oxygen atoms of the two hydroxyl groups of AB4 formed two π-H bonds with TyrC56 and TyrA56 (Fig. 8A). In addition, the hydroxyl groups of aesculin formed two hydrogen bonds with TyrC56 and LysA124 acting as H-acceptors (Fig. 8A). Similarly, the phenyl ring of benzopyran-2-one of aesculin formed π-H bonds with residue AspA122 of PD-L1 (Fig. 8A). Furthermore, all six components in *R. coptidis* and/or *C. phellodendri* components, which are isoquinoline alkaloids, bound to PD-L1 through interactions with their isoquinoline rings (Fig. 8B); this binding mode was similar to that of BMS-202. Four of these compounds, namely berberine, jatrorrhizine, coptisine, and phellodendrine, formed π-H bonds with residues TyrC56, AlaA121, and/or AspA122 of PD-L1; this interaction occurred through the phenyl ring or the protonated amine of isoquinoline ring of the compounds (Fig. 8B). In addition, palmatine, coptisine, and epiberberine formed metal contacts with AspA122. These results suggest that *R. coptidis* and/or *C. phellodendri* contain several potential PD-L1 ligands.

### Verification of the in-vitro anti-inflammatory activity of the identified immunomodulators from BTWT immunomodulatory components of BTWT

**Fig. 9:**
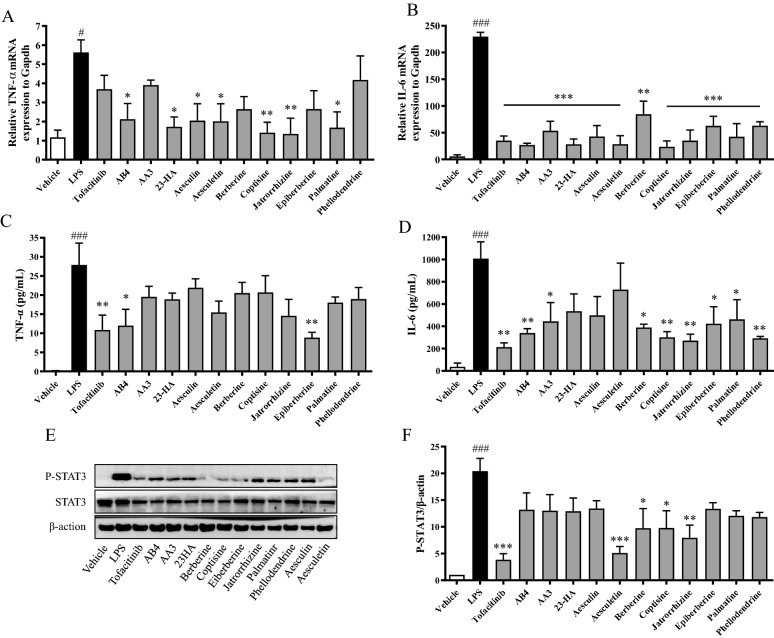
The effects of the identified immunomodulators from BTWT on inflammatory responses in vitro. The effects of BTWT components on the production of cytokines in LPS-stimulated RAW264.7 cells were assessed by RT-qPCR (**A** and **B**) and ELISA (**C** and **D**), and the effects on STAT3 activation were assessed by western blot (**E** and **F**). ^#^P < 0.05 and ^###^P < 0.001 compared with the vehicle control; *P < 0.05, **P < 0.01, and ***P < 0.001 compared with the LPS group (n = 3)

The effects of the BTWT components (showing no cytotoxicity on cultured splenocytes of mice at a concentration of 1 μM, Additional file 1: Fig. S2) on inflammatory responses were investigated using cultured RAW 264.7 cells. LPS stimulation of RAW 264.7 cells increased mRNA and protein levels of TNF-α and IL-6 (Fig. 9A–D). Importantly, the TNF-α and IL-6 levels in RAW 264.7 cells pretreated with the 11 compounds were much lower than those in vehicle-treated controls (Fig. 9A–D). All the compounds significantly reversed the LPS-mediated increase in p-STAT3 expression, and this effect was similar to that produced by tofacitinib (Fig. 9E, F).

## Discussion

UC is a chronic, relapsing inflammatory disease of the gastrointestinal tract that has heterogeneous symptoms and prognosis. Up to 15% of UC patients require surgery and an increased duration of disease results in a high risk of developing colorectal cancer, especially in Asian and North American patients [31]. The incidence and prevalence of UC are 1.2–20.1 and 7.6–245 cases per 100,000 person/year worldwide, respectively, and these numbers continue to rise [46–49], resulting in a huge economic burden and increasing healthcare costs [50, 51]. Unfortunately, there is no satisfactory treatment; corticosteroids have long been used to suppress the inflammation associated with the condition. New discoveries concerning the underlying mechanisms involved in the pathogenesis of UC have enabled new treatment options to be developed. Small molecules have several advantages over biological therapies, including their lack of immunogenicity, short half-life, oral administration, and low manufacturing cost [52]. Among small-molecule strategies, JAK inhibitors have emerged as a novel approach to modulate downstream cytokine signaling during immune-mediated diseases [30]. Tofacitinib is an oral, non-selective JAK inhibitor that inhibits the JAK-STAT signaling pathway and thereby inhibits the inflammatory response [53]. It was approved by the US Food and Drug Administration for treating severe rheumatoid arthritis in November 2012 [54] and for treating moderate to severe UC in May 2018, but the clinical remission rate for the treatment of UC is only about 40% [55]. S1PR agonism is another novel strategy for combating autoimmune diseases that acts, in part, by interfering with lymphocyte trafficking by blocking lymphocyte egress from lymph nodes [16]. Small-molecule S1PR modulators bind to S1PR1, which leads to irreversible internalization and degradation of the receptor, which eventually causes functional antagonism [56]; through this mechanism, S1PR modulators block S1PR1-mediated lymphocyte migration from lymph nodes to inflammatory organs. Fingolimod is the first approved S1PR1 regulator and is used to treat multiple sclerosis [57]. Several S1PR1 modulators, such as etrasimod, are currently being investigated as a new class of drugs for treating UC [58]. Indeed, the anti-UC effect of fingolimod in mice with relapsing colitis was shown in our previous work [29].

TCM-derived compounds provide new resources for identifying small molecules that can be used in UC; such compounds will have greatly reduced development costs as most of them can be easily isolated from herbs [59, 60]. BTWT has been widely used in China for thousands of years and is currently used for the clinical treatment of UC [1]. It reduces the levels of inflammatory cytokines such as IL-1β, IL-6, and TNF-α in the serum of UC mice [2] and regulates the balance between Th17 and Treg cells [61]. In addition, BTWT has anti-inflammatory effects and may act as an immunomodulator in a mouse model of LPS-induced systemic inflammation [62]. However, prior to this study, it was largely unknown which active components of BTWT were responsible for its immunomodulatory activity.

In our study, commercial concentrated granules were used as an alternative to a decoction to obtain a BTWT formula. Individual ingredients known to constitute the raw materials of the BTWT (that is, standards) were analyzed using the same chromatogram conditions as those used to confirm the peaks obtained from the LC–MS analysis of the BTWT granule constituents. A total of 11 compounds were identified and assigned to an individual known ingredient. After verification of the quality of the components of the BTWT granules, 531.5 mg/kg of material isolated from BTWT granules was used in both the preventative and therapeutic strategies to treat DSS-induced acute or chronic colitis in mice. This amount of BTWT had a pronounced protective effect in reducing the DAI and inhibiting colon shortening and swelling. In addition, BTWT granule extract inhibited colonic inflammation by suppressing cytokine production, including TNF-α, IL-6, and IL-17. These observations indicate that BTWT exerts immunomodulatory activities and protects against UC in animal models.

To define the absorbed chemical constituents, the comparison of mass spectrometry data from compounds distributed in the circulation and/or colon tissue of mice was compared with that of standard chemicals. We demonstrated that three compounds, 23-HA, epiberberine, and aesculetin, were only detected in colon samples. The remaining eight components, AB4, AA3, berberine, palmatine, coptisine, jateorhizine, phellodendrine, and aesculin, were detected in both colon and plasma samples. These observations indicate that all 11 components of BTWT could be absorbed and distributed in either the circulation or the target tissue (the colon) to exert biological activities. Thus, in subsequent docking studies, all 11 compounds contained in BTWT were used to identify potential immunomodulators against six reported pharmacological targets relevant to UC.

As summarized in Table 1, the comparison of the docking of positive-control ligands and test compounds enabled the identification of two S1PR1 (AB4 and AA3) ligands, two STAT3 (AA3 and 23-HA) ligands, and one PD-1/PD-L1 (AB4) ligand from the compounds contained in *R. pulsatilla*. Four components of *R. coptidis/C. phellodendri* components were identified as ligands for JAK3, six components were identified as ligands for PD-L1, and one component (obakunone) was a ligand for PD-1. Furthermore, the two components of *C. fraxini* were identified as ligands for JAK3, and aesculin was identified as a ligand for STAT3, S1PR1 and PD-L1.

**Table 1:** The components of BTWT that bound to five selected protein targets in a similar way as positive-control compounds

BTWT	Components	JAK3	STAT3	S1PR1	PD-1	PD-L1
*R. pulsatilla*	AB4			√	√	√
	AA3		√	√		
	23-HA		√			
*R. coptidis*/ *C. phellodendri*	Berberine	√				√
	Palmatine	√			√	√
	Jatrorrhizine				√	√
	Coptisine	√				√
	Epiberberine	√			√	√
	Phellodendrine				√	√
*C. fraxini*	Aesculin	√	√	√		√
	Aesculetin	√				

AB4 is the major active component of *Pulsatilla* decoction, and its therapeutic potential has been reviewed by us and is shown to be a promising compound for treating inflammatory diseases, especially UC [63]. However, the precise mechanisms of action of AB4 are largely unknown; nevertheless, a recent study verified that AB4 prevented symptoms in an animal model of acute UC and inhibited the TLR4/NF-κB/MAPK signaling pathway [64]. Thus, our present study is the first to demonstrate that AB4 is a potential immunomodulator by targeting S1PR1 and PD1/PDL1. AA3 and 23-HA are likely the active metabolites of AB4, as hypothesized in our recent review [63]. Due to the structural relationship between AB4 and AA3, it is rational to assume that AA3 is also an S1PR1 modulator, and indeed we demonstrated this in the current study. More interestingly, AA3 and 23-HA are potential immunomodulators through their interactions with STAT3. The current results are consistent with those of our previous study, in which we showed that AA3 protects against neuronal inflammatory disease by reducing the infiltration of Th17 cells into the spinal cord by inhibiting STAT3 activation [65].

The alkaloids contained in *R. coptidis and C. phellodendri* formed several π-H interactions with each tested protein; the interaction between their isoquinoline structure and JAK3 or PD-L1 was similar to the interaction observed with the binding of the respective positive-control ligands to JAK3 or PD-L1. Berberine is the most abundant component of *C. phellodendri* and *R. coptidis*, and as such represents an important component of *Pulsatilla* decoction that is a promising candidate for UC treatment, as shown by us [27–29]. In the present study, we demonstrated that berberine formed a π-H bond with Leu828 of JAK3. Interestingly, a previous study reported that berberine is selective for JAK3 over other JAK kinase members in various cellular assays and forms hydrogen bonds with the kinase domain of JAK3, as demonstrated using AutoDock version 4 [66]. These observations strongly suggest that berberine exerted anti-UC activity by targeting JAK3, although the precise binding sites need further verification. In addition, we observed that the phenyl ring of berberine formed a π-H bond with TyrC56 of PD-L1, similar to that observed with the positive-control ligand. A previous study reported that the deubiquitination activity of berberine resulted in the selective ubiquitination and degradation of PD-L1, which inhibited the activity of PD-1/PD-L1 [67]. Whether berberine acts as a direct modulator of PD-L1 remains to be clarified. Furthermore, the five components of *C. phellodendri* and *R. coptidis* that have a similar structure to berberine and similar binding modes are also potential ligands for JAK3, PD1, or PDL1. These observations suggest that these components could also have anti-UC activity, similar to that observed with berberine, palmatine, and coptisine [11–13].

Aesculin and aesculetin are the label ingredients of *R. coptidis* and have anti-inflammatory activities in vivo [14]. In our present study, we demonstrated that aesculin is a ligand for several protein targets relevant to UC treatment. This observation suggests that aesculin is a promising anti-UC agent, and is consistent with findings showing that aesculin relieves symptoms of DSS-induced colitis and restricts the expression of inflammatory factors [14]. The other component of *R. coptidis*, aesculetin, is structurally similar to aesculin and both have a similar binding profile to JAK3, STAT3, or S1PR1, and so could also be potential anti-UC agents. Whether aesculetin shows activity against UC and modulates the JAK/STAT pathway needs to be further explored. Nevertheless, we showed that the component of BTWT could act as immunomodulators by showing that they had anti-inflammatory actions in cultured RAW 264.7 cells.

In summary, in combination with the efficacy and absorbed constituent study, the *in-silico* docking simulation allowed us to identify the components of BTWT that bind to protein targets that are known to be involved in the pathogenesis of UC or are therapeutic targets for clinically used drugs. These findings suggest that these identified components act as immunomodulators, and that the mechanism of action of *Pulsatilla* decoction on UC involves multiple targets. Future systematic assessment of the targeting of these components to the respective proteins will be conducted to better clarify the mechanism of action of each component.

## Supplementary Information

**Additional file 1: Fig. S1.** M Molecular docking results of i) the positive-control ligand tofacitinib in the binding sites of JAK3, ii) SD-36 in the binding sites of STAT3, iii) S1P and ML056 (W146) in the binding sites of S1PR1 (A), iv) ML385 in the binding sites of Nrf2, nivolumab in the binding sites of PD-1, and v) BMS-202 in the binding sites of PD-L1 (B) with the lowest calculated binding energy conformations. In the 2D images, the polar and nonpolar residues of the active site of the proteins are shown in purple and green, respectively. Hydrogen bonds in the sidechain and backbone are shown in red and blue line arrows, respectively; the arrows point to the H-acceptor. The π-H interactions are marked with a red line and an arene-H label. The metal contact interactions are shown with a purple line. In the 3D images, the carbon atoms of the compounds are shown in yellow, and other atoms (e.g., oxygen, nitrogen, and sulfur atoms) are shown in blue. The amino acid residues of the proteins that interacted with the compounds are shown in green. The formation of hydrogen bonds, π-H interactions, and π-π interactions are shown by a red dotted line. **Fig. S2.** The effects of 11 components of BTWT on cell viability of splenocytes assessed by MTT assay after 24 h treatment (n = 3).

## Data Availability

All data generated or analyzed during this study are included in this published article [and its supplementary information files].

## Abbreviations

BTWT: Bai-Tou-Weng-Tang
TCM: Traditional Chinese medicine
UC: Ulcerative colitis
*R pulsatilla*: *Radix pulsatilla *
*C. Phellodendri*: *Cortex phellodendri*
*R. coptidis*: *Rhizoma coptidis*
*C. fraxini*: *Cortex fraxini*
AB4: Anemoside B4
AA3: Anemoside A3
DSS: Dextran sulfate sodium
LPS: Lipopolysaccharide
JAK: Janus tyrosine kinase
STAT: Signal transducer and activator of transcription
S1PR: Sphingosine-1-phosphate receptor modulator
PD-1: Programmed cell death protein 1
PD-L1: Programmed cell death protein ligand 1
Nrf2: Nuclear factor-erythroid2-related factor 2
DAI: Disease activity index
TNF-α: Tumor necrosis factor α
IL-6: Interleukin 6
IL-17: Interleukin 17
RT-qPCR: Real-time reverse-transcription polymerase chain reaction
