# Supplementary material for: Identification of potential immunomodulators from Pulsatilla decoction that act on therapeutic targets for ulcerative colitis based on pharmacological activity, absorbed ingredients, and in-silico molecular docking
Source: Chin Med. 2022 Nov 24;17:132. doi: 10.1186/s13020-022-00684-7 (PMC9701001; doi:10.1186/s13020-022-00684-7)
Supplement: Supplementary file 1 — Additional file 1: Fig. S1. M Molecular docking results of i) the positive-control ligand tofacitinib in the binding sites of JAK3, ii) SD-36 in the binding sites of STAT3, iii) S1P and ML056 (W146) in the binding sites of S1PR1 (A), iv) ML385 in the binding sites of Nrf2, nivolumab in the binding sites of PD-1, and v) BMS-202 in the binding sites of PD-L1 (B) with the lowest calculated binding energy conformations. In the 2D images, the polar and nonpolar residues of the active site of the proteins are shown in purple and green, respectively. Hydrogen bonds in the sidechain and backbone are shown in red and blue line arrows, respectively; the arrows point to the H-acceptor. The π-H interactions are marked with a red line and an arene-H label. The metal contact interactions are shown with a purple line. In the 3D images, the carbon atoms of the compounds are shown in yellow, and other atoms (e.g., oxygen, nitrogen, and sulfur atoms) are shown in blue. The amino acid residues of the proteins that interacted with the compounds are shown in green. The formation of hydrogen bonds, π-H interactions, and π-π interactions are shown by a red dotted line. Fig. S2. The effects of 11 components of BTWT on cell viability of splenocytes assessed by MTT assay after 24 h treatment (n = 3). [file 13020_2022_684_MOESM1_ESM.pdf]

Fig. S1

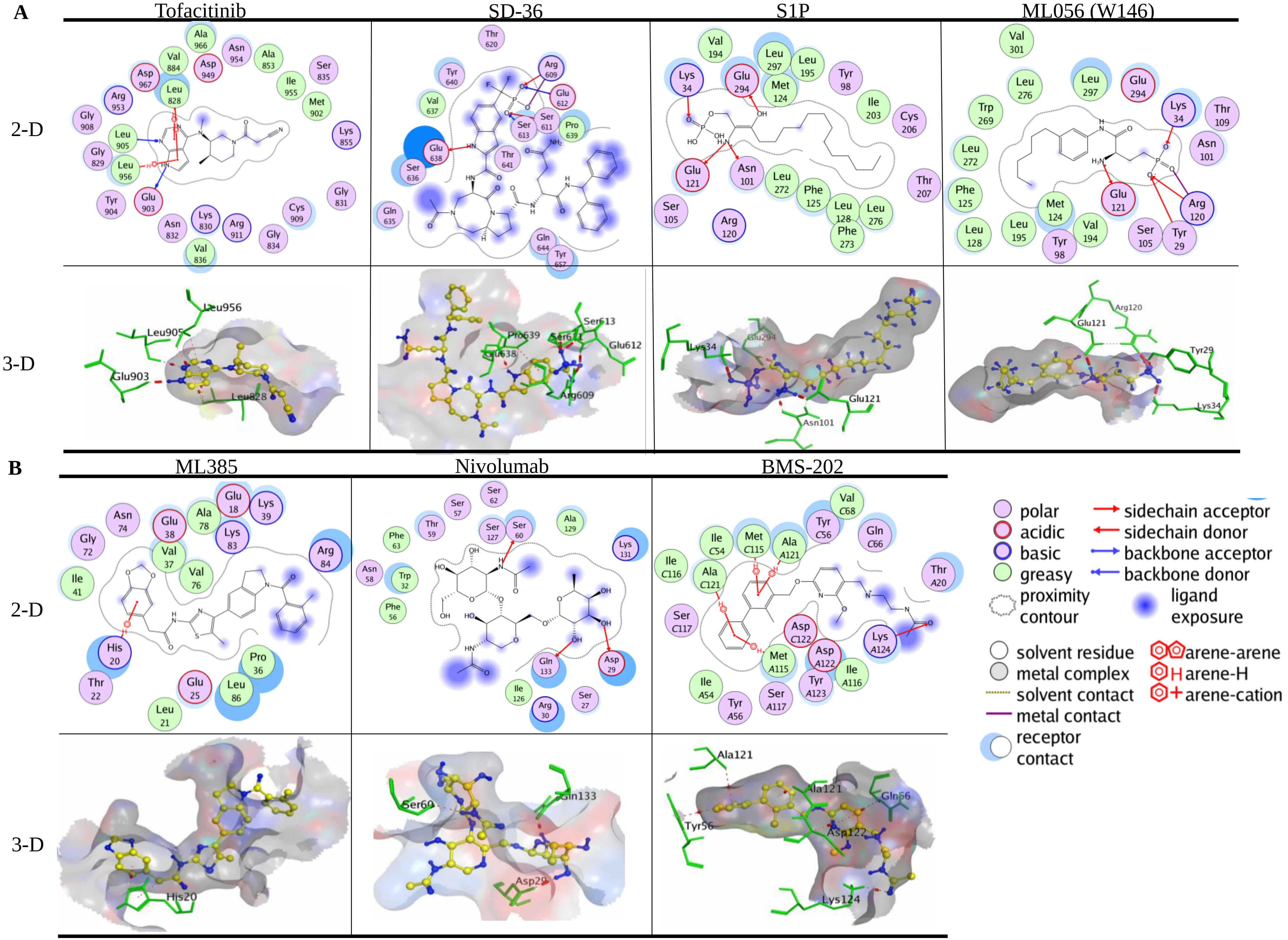
A
Tofacitinib
SD-36
S1P
ML056 (W146)
2-D
3-D
B
ML385
Nivolumab
BMS-202
2-D
3-D
polar
acidic
basic
greasy
proximity contour
sidechain acceptor
sidechain donor
backbone acceptor
backbone donor
ligand exposure
solvent residue
metal complex
solvent contact
metal contact
receptor contact
arene-arene
arene-H
arene-cation

Fig. S2

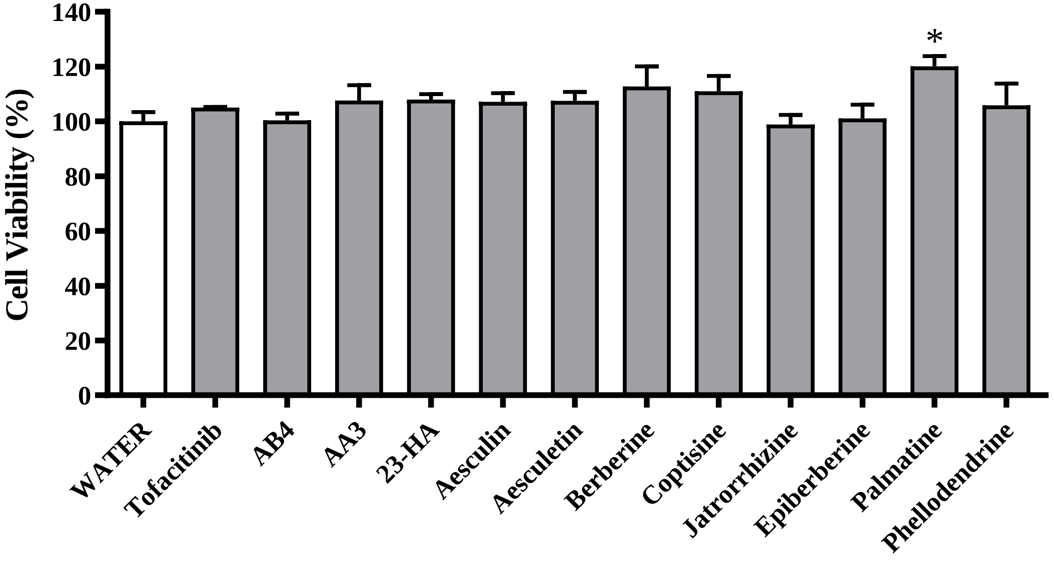

Cell Viability (%)
140
120
100
80
60
40
20
0
*
WATER
Tofacitinib
AB4
AA3
23-HA
Aesculin
Aesculetin
Berberine
Coptisine
Jatrorrhizine
Epiberberine
Palmatine
Phellodendrine
